# Anterior chamber angle imaging with swept-source optical coherence tomography: comparison between CASIAII and ANTERION

**DOI:** 10.1038/s41598-020-74813-3

**Published:** 2020-10-30

**Authors:** Poemen Pui-man Chan, Gilda Lai, Vivian Chiu, Anita Chong, Marco Yu, Christopher Kai-shun Leung

**Affiliations:** 1 Department of Ophthalmology & Visual Sciences, The Chinese University of Hong Kong, Hong Kong Eye Hospital, Kowloon, Hong Kong, People’s Republic of China; 2 Hong Kong Eye Hospital, Hong Kong, SAR People’s Republic of China; 3 Singapore Eye Research Institute, Singapore National Eye Centre, Singapore, Singapore

**Keywords:** Glaucoma, Diagnostic markers

## Abstract

This study compared the test–retest variabilities and measurement agreement of anterior chamber angle (ACA) dimensions measured by two anterior segment swept-source optical coherence tomography (SS-OCT)—the ANTERION (Heidelberg Engineering, Heidelberg, Germany) and CASIAII (Tomey, Nagoya, Japan). Thirty-eight subjects, 18 patients with primary angle closure and 20 healthy participants with open angles, were included. The mean age was 54.7 ± 15.8 years (range: 26–75 years). One eye of each subject was randomly selected for anterior segment imaging by ANTERION and CASIAII, using the same scan pattern (6 evenly spaced radial scans across the anterior segment for three times) in the same visit. The between- and within-instrument agreement and repeatability coefficients of angle open distance (AOD500), trabecular-iris space area (TISA500), lens vault (LV), scleral spur-scleral spur distance (SSD), anterior chamber depth (ACD), and pupil diameter (PD) were measured. The anterior and posterior boundaries of the cornea, iris, and lens were automatically segmented by the SS-OCT instruments; the scleral spur was manually located by a single masked observer. There were significant differences between ANTERION and CASIAII measurements; the SSD, PD, and ACD were smaller whereas AOD500 and TISA500 were greater in ANTERION compared with CASIAII (*P* < 0.001). Anterior segment measurements obtained from the two SS-OCT instruments showed strong associations (R^2^ ranged between 0.866 and 0.998) although the between-instrument agreement was poor; the spans of 95% limits of between-instrument agreement were ≥ 1.5-folds than the within-instrument agreement for either instrument. Whereas both SS-OCT instruments showed low test–retest measurement variabilities, the repeatability coefficients of AOD500, TISA500, ACD, and PD were slightly smaller for CASIAII than ANTERION (P ≤ 0.012).

## Introduction

Assessment of the anterior chamber angle (ACA) dimensions and the anterior chamber depth (ACD) is essential in the diagnostic evaluation of primary angle closure disease (PACD). Although gonioscopy and slit-lamp biomicroscopy are not precise enough for reliable measurements of the anterior segment structures, anterior segment optical coherence tomography (AS-OCT) has enabled non-contact acquisition of cross-sectional anterior segment images for evaluation of the ACD, lens vault (LV), and the ACA dimensions in the dark^1–6^. The CASIAII (Tomey, Nagoya, Japan), introduced in 2016–2017, is the second generation of swept-source anterior segment optical coherence tomography (AS-OCT). Different from the first generation (CASIA SS-1000), it offers a faster scan-speed (50,000 vs. 30,000 A-scans/s) and a higher transverse resolution (800 A-scans/B-scan vs. 256 A-scans/B-scan) for 360° imaging of the ACA using 18 evenly-spaced radial scans over 36 angle locations. Introduced in 2019, the ANTERION (Heidelberg Engineering, Heidelberg, Germany) represents another swept-source AS-OCT technology which also allows axial length measurement for ocular biometry in addition to anterior segment imaging. The “Metrics App” measures the ACA dimensions in 6 evenly-spaced radial scans over 12 angle locations. Whether the two swept-source AS-OCT instruments have comparable ACA measurements and test–retest variabilities remains unclear. In this study, we evaluated the between- and within-instrument agreement, and compared the test–retest variabilities of ACD, LV, and ACA measurements obtained from CASIAII and ANTERION.

## Methods

### Subjects

A total of 38 eyes of 38 subjects (20 healthy individuals and 18 patients with PACD) were consecutively recruited from June to July 2019 at the University Eye Center, Hong Kong Eye Hospital. Patients with PACD had primary angle-closure suspect (PACS) or primary angle closure (PAC). Healthy individuals had open-angles in dark-room gonioscopy and unremarkable anterior and posterior segment examination. Patients with PACS had posterior trabecular meshwork invisible for ≥ 180° in dark-room gonioscopy; patients with PAC had intraocular pressure (IOP) measured with Goldmann applanation tonometry > 21 mmHg on at least two separate visits in addition to angle closure. Gonioscopy was performed with a 4-mirror gonioprism at the lowest level of ambient illumination that permitted a view of the angle at 16X to 25X magnification. One eye of each participant was randomly selected for AS-OCT imaging; three separate datasets (each dataset comprised 6 evenly-spaced radial B-scans) were obtained from each of the two AS-OCT instruments—CASIAII (Tomey) and ANTERION (Heidelberg Engineering)—in the same visit. Two datasets with clear scleral spur in the B-scans obtained from the individual AS-OCT instruments were analyzed. The study was conducted in accordance with the ethical standards stated in the 2013 Declaration of Helsinki and approved by Hong Kong Kowloon Central Research Ethics Committee with written informed consent obtained.

### Anterior segment optical coherence tomography imaging

The CASIAII (Tomey, Nagoya, Japan) used a monochromatic tunable fast scanning laser source and a photodetector to detect wavelength-resolved interference signal. The original scan protocol for ACA imaging comprised 18 evenly-spaced, radial B-scans but we modified the scan protocol to provide 6 evenly-spaced radial B-scans, similar to that in ANTERION (Table 1). For CASIAII, each B-scan had 800 A-scans; for ANTERION, each B-scan had 768 A-scans. All eyes were imaged in the dark (light intensity, 0.3 lx). For both AS-OCT instruments, the subjects were asked to fixate at an internal fixation target. To avoid lid artifact, the technician would retract the upper and lower lids of the participant while taking caution not compressing the globe. All images obtained had no lid artifacts.

**Table 1:** Specifications and scan protocols of anterior chamber angle imaging for ANTERION and CASIAII.

	ANTERION (Metrics App)	CASIAII
Light source wavelength (nm)	1300	1310
Axial resolution (µm)	< 10	< 10
Transverse resolution (µm)	< 30	< 30
Scan speed (A-scans per second)	50,000	50,000
Scan depth (mm)	14 ± 0.5	13
Maximum scan width (mm)	16.5	16
Scan pattern	6 evenly-spaced radial B-scans	6 evenly-spaced radial B-scans^a^
Number of A-scans per B-scan	768	800

^a^Custom-designed for the study.

### Measurements of the anterior chamber angle

The CASIAII and ANTERION had built-in interface for measurements of the angle opening distance (AOD), trabecular iris space area (TISA), ACD, lens vault (LV), scleral spur to scleral spur distance (SSD), and pupil diameter (PD). The anatomic boundaries including the anterior corneal surface, posterior corneal surface, anterior iris surface, posterior iris surface, anterior lens surface, and posterior lens surface were automatically segmented from the respective OCT instruments. The scleral spur was manually located by a single masked observer for measurements of the AOD/TISA measurements, SSD, and LV. Scleral spur was identified from the point of inward protrusion of the sclera at the interface between the less reflective ciliary muscle and the more reflective corneoscleral junction at the inner corneal margin^7^. AOD500 was the perpendicular distance between anterior iris surface and a point at the trabecular meshwork 500 µm anterior to the scleral spur^8^. TISA500 referred to the area bounded by the AOD500, inner corneoscleral wall, iris surface and a line drawn from the scleral spur, that is perpendicular to the plane of the inner scleral wall, to the opposing iris^9^. AOD500 and TISA500 were measured every 30° for 12 angle locations. The angle locations were annotated with right eye orientation with 0° denoting the nasal angle and 180° denoting the temporal angle. The SSD was the shortest distance joining the scleral spurs^5^; PD was the shortest distance joining the pupil margin; ACD was the distance from the endothelial surface of the corneal apex to the anterior surface of the crystalline lens; LV was the perpendicular distance between the anterior pole of the crystalline lens and the horizontal line joining the two scleral spurs opposite of each other^10^. The mean SSD, PD, ACD, and LV were calculated from taking the average from the 6 B-scans in an eye.

## Statistics

Statistical analyses were performed using MedCalc 19.0.7 (MedCalc Software, Mariakerke, Belgium) and Excel (Office 365, Microsoft USA). Demographics and the anterior segment parameters between the normal and PACD eyes were compared with independent *t*-test. Bland–Altman plot was used to report the measurement agreement between the two OCT instruments (each subject had one dataset from the respective instruments for the agreement analysis). Repeatability coefficient (RC) was defined as 1.96 x √2 × S_W_, of which S_W_ is the within-subject standard deviation. The difference between the two measurements of the same eye is expected to be less than 1.96 x √2 × S_W_ for 95% of pairs of observations^11^. The current sample size would give a confidence interval of 22.5% either side of the estimate S_W_ for 2 observations per subject. Comparison of RCs between the two measurements was evaluated by empirical bootstrap t test with 2000 replicates. The association of anterior segment parameters measured by the two OCT instruments were evaluated with linear regression analysis.

## Results

The demographics of the normal group and the PACD group are shown in Table 2. Patients with PACD were older, had larger LV, and smaller axial length, ACD, SSD, PD, AOD500 and TISA500 measurements compared with healthy individuals (P ≤ 0.034).

**Table 2:** Demographics and anterior segment optical coherence tomography measurements (mean ± SD) of the normal and primary angle closure disease (PACD) groups.

	Normal group (Mean ± SD) (n = 20)	PACD group (Mean ± SD) (n = 18)	P*
Age (years)	46.35 ± 17.46	64.00 ± 5.52	< 0.001
Intraocular pressure (mmHg)	14.95 ± 2.80	16.26 ± 4.30	0.256
Axial Length (mm)	24.93 ± 1.12	23.10 ± 0.78	< 0.001
Dark-room gonioscopy grading (Shaffer)	3.55 ± 0.69	0.64 ± 0.84	< 0.001
**ANTERION**			
Anterior chamber depth (mm)	2.91 ± 0.44	2.00 ± 0.33	< 0.001
Pupil diameter (mm)	5.60 ± 1.10	4.59 ± 0.93	0.003
Scleral spur-scleral spur distance (mm)	11.79 ± 0.34	11.58 ± 0.28	0.004
Lens vault (mm)	0.176 ± 0.332	0.979 ± 0.282	< 0.001
Angle opening distance 500 (mm)	0.452 ± 0.227	0.109 ± 0.086	< 0.001
Trabecular iris space area 500 (mm^2^)	0.149 ± 0.072	0.044 ± 0.025	< 0.001
**CASIAII**			
Anterior chamber depth (mm)	2.95 ± 0.44	2.04 ± 0.33	< 0.001
Pupil diameter (mm)	5.73 ± 1.08	4.77 ± 1.10	0.010
Scleral spur-scleral spur distance (mm)	11.82 ± 0.33	11.60 ± 0.27	0.034
Lens vault (mm)	0.178 ± 0.329	0.986 ± 0.281	< 0.001
Angle opening distance 500 (mm)	0.426 ± 0.220	0.093 ± 0.087	< 0.001
Trabecular iris space area 500 (mm^2^)	0.145 ± 0.070	0.034 ± 0.030	< 0.001

*Independent t-test. OCT measurements were derived from the mean of 6 evenly-spaced radial scan measurements.

### Comparisons of anterior segment measurements between ANTERION and CASIAII

**Figure 1:**
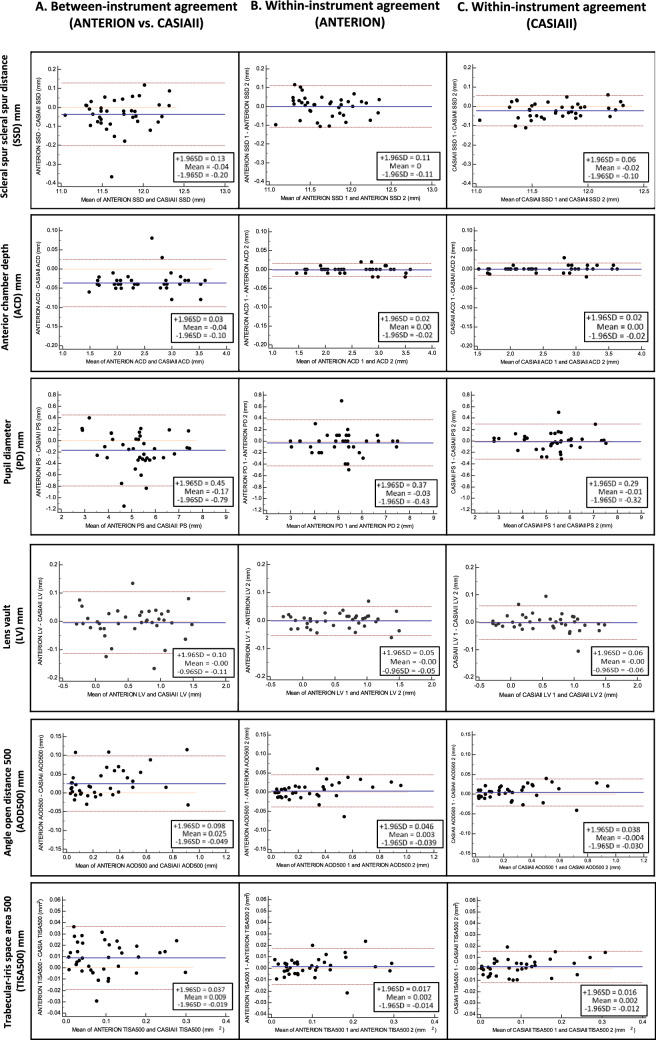
Bland–Altman plot: comparison of different anterior segment parameters. (**A**) Between-instrument agreement (ANTERION vs. CASIAII). (**B**) Within-instrument agreement (ANTERION). (**C**) Within-instrument agreement (CASIAII).

**Figure 2:**
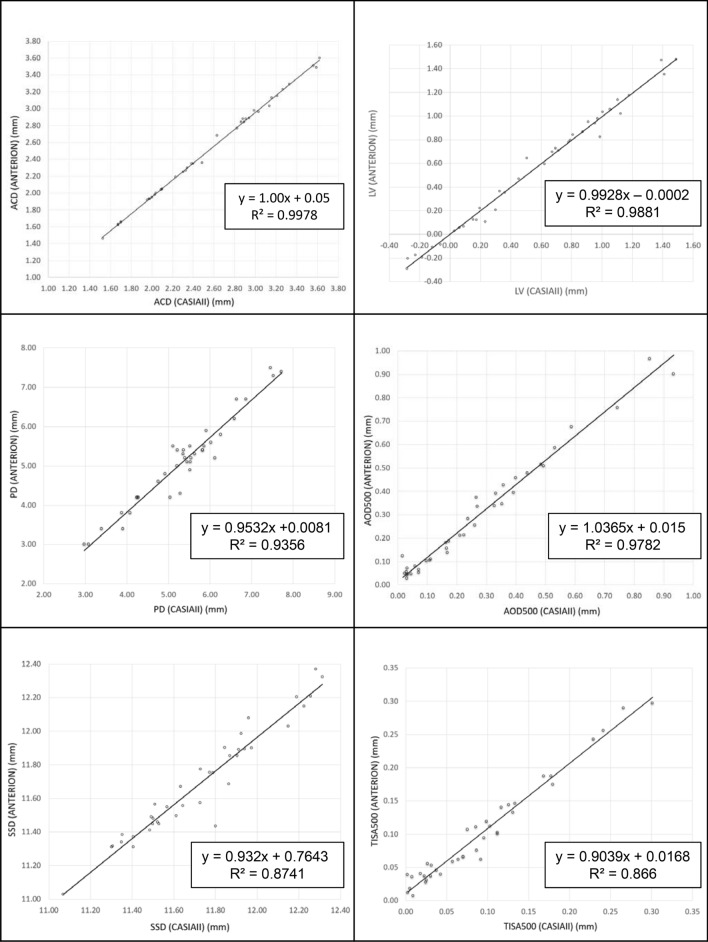
Regression analysis of the anterior segment parameters measured from ANTERION and CASIAII. ACD, anterior chamber depth; PD, pupil diameter; SSD, scleral spur-scleral spur distance; LV, lens vault; angle open distance 500 (AOD500); trabecular-iris space area 500 (TISA500).

**Figure 3:**
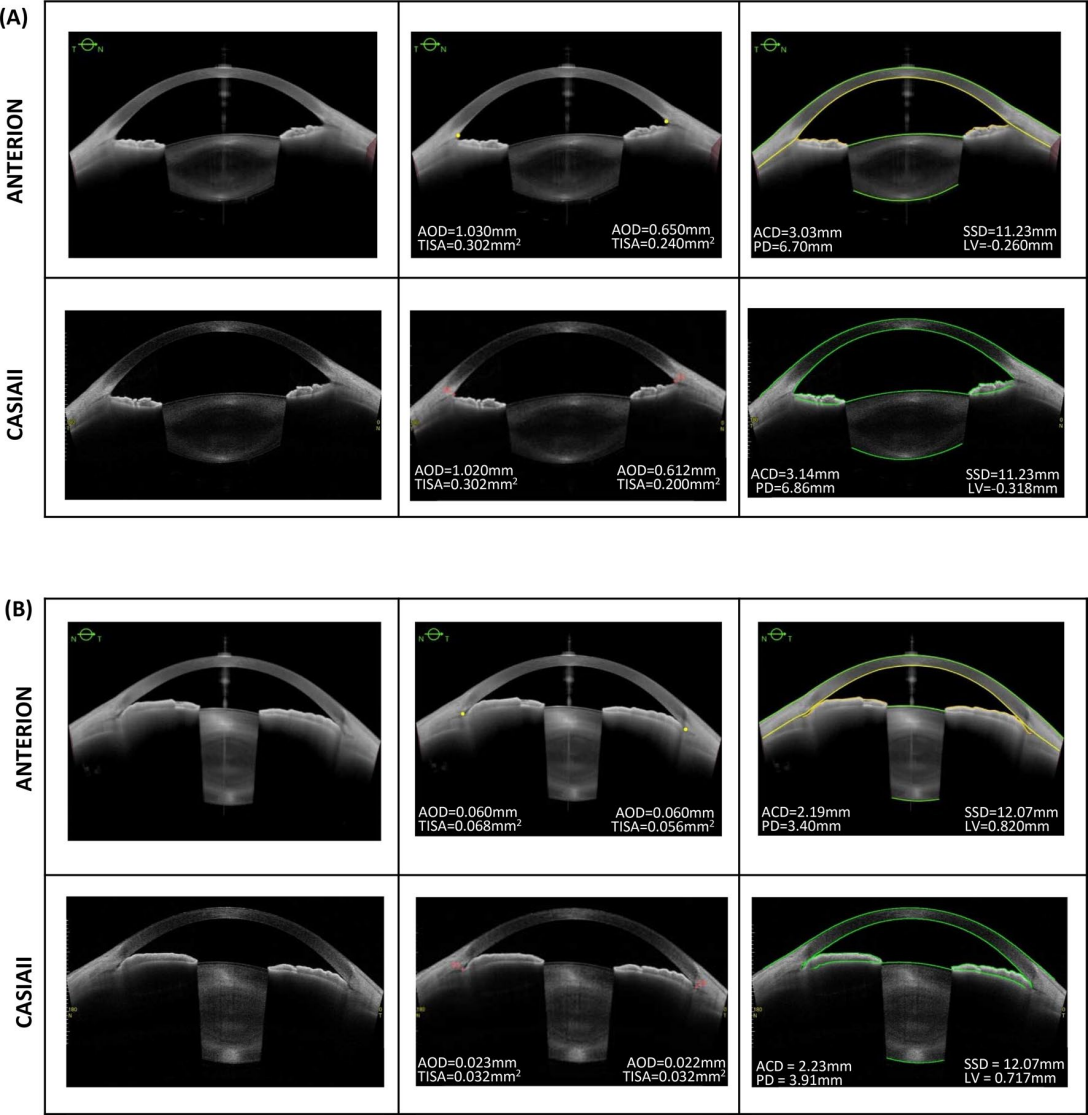
An eye with open angle (**A**) and an eye with primary angle closure (**B**) imaged by the ANTERION and CASIAII. The scleral spur locations were manually marked (middle panel); the anterior and posterior boundaries of the cornea and the lens were automatically segmented by the respective instruments (right panel). ACD, anterior chamber depth; PD, pupil diameter; SSD, scleral spurs distance; LV, lens vault; AOD, angle opening distance at 500 µm; TISA, trabecular iris space area at 500 µm. Only the horizontal scan (0°–180°) are shown.

ANTERION showed smaller mean SSD (11.69 ± 0.36 mm vs. 11.73 ± 0.31 mm, p < 0.001), ACD (2.48 ± 0.60 mm vs. 2.51 ± 0.60 mm, p < 0.001), and PD (5.11 ± 1.12 mm vs. 5.36 ± 1.17 mm, p < 0.001) compared with CASIAII (Table 3). The mean LV measured by ANTERION was also smaller than that measured by CASIAII although the difference was not statistically significant (p = 0.639). The mean AOD500 and TISA500 measured by ANTERION, however, were greater than those measured by CASIAII (p < 0.001). The between-instrument agreement of AOD500, TISA500, ACD, LV, PD, SSD measurements was poor (Fig. 1A); the spans of 95% limits of agreement were 0.147 mm, 0.056 mm^2^, 0.13 mm, 0.21 mm, 1.24 mm, and 0.33mm, respectively, which were considerably greater (≥ 1.5-fold) than the within-instrument agreement of the corresponding parameters for both ANTERION (0.085 mm, 0.031 mm^2^, 0.04 mm, 0.10 mm, 0.80 mm, and 0.22mm, respectively) (Fig. 1B) and CASIAII (0.068 mm, 0.028mm^2^, 0.04 mm, 0.12 mm, 0.61 mm, and 0.16mm, respectively) (Fig. 1C). The strength of association of all the anterior segment parameters between ANTERION and CASIAII was strong; the R^2^ ranged between 0.866 and 0.998 (Fig. 2). Figure 3 shows examples of images taken by the ANTERION and CASIAII for eyes with open angle and narrow angle, respectively.

**Table 3:** Comparisons of anterior segment parameters (mean ± SD) between ANTERION and CASIAII (n = 38 eyes; 20 healthy eyes with open-angles and 18 eyes with PACD).

Clock-hours	Anterior chamber depth, ACD (mm)			Pupil diameter, PD (mm)		
	ANTERION	CASIAII	*P*	ANTERION	CASIAII	*P*
Mean	2.48 ± 0.60	2.51 ± 0.60	< 0.001	5.11 ± 1.12	5.36 ± 1.17	< 0.001

Clock-hours	Scleral spur—scleral spur distance, SSD (mm)			Lens vault, LV (mm)		
	ANTERION	CASIAII	*P*	ANTERION	CASIAII	*P*
Mean	11.69 ± 0.36	11.73 ± 0.31	< 0.001	0.556 ± 0.508	0.561 ± 0.509	0.639
1, 7	11.76 ± 0.34	11.81 ± 0.34	0.003	0.571 ± 0.499	0.568 ± 0.501	0.796
2, 8	11.60 ± 0.32	11.62 ± 0.33	0.427	0.509 ± 0.528	0.513 ± 0.521	0.647
3, 9	11.59 ± 0.34	11.57 ± 0.32	0.402	0.528 ± 0.539	0.513 ± 0.530	0.078
4, 10	11.55 ± 0.32	11.60 ± 0.30	0.025	0.527 ± 0.510	0.521 ± 0.519	0.586
5, 11	11.77 ± 0.35	11.80 ± 0.33	0.183	0.587 ± 0.497	0.610 ± 0.523	0.259
6, 12	11.88 ± 0.36	11.95 ± 0.38	0.011	0.626 ± 0.481	0.646 ± 0.481	0.106

Clock-hours	Angle opening distance at 500 µm, AOD500 (mm)			Trabecular iris space area at 500 µm, TISA500 (mm^2^)		
	ANTERION	CASIAII	*P*	ANTERION	CASIAII	*P*
Mean	0.291 ± 0.254	0.267 ± 0.250	< 0.001	0.100 ± 0.080	0.092 ± 0.082	< 0.001
1	0.244 ± 0.216	0.202 ± 0.177	< 0.001	0.085 ± 0.066	0.074 ± 0.060	0.003
2	0.267 ± 0.237	0.233 ± 0.191	0.018	0.096 ± 0.069	0.086 ± 0.065	0.020
3	0.307 ± 0.258	0.270 ± 0.224	0.002	0.110 ± 0.086	0.097 ± 0.074	0.006
4	0.308 ± 0.275	0.287 ± 0.260	0.042	0.100 ± 0.084	0.100 ± 0.088	0.930
5	0.270 ± 0.257	0.260 ± 0.277	0.475	0.095 ± 0.081	0.092 ± 0.096	0.579
6	0.271 ± 0.240	0.231 ± 0.219	0.004	0.094 ± 0.076	0.073 ± 0.068	< 0.001
7	0.302 ± 0.263	0.298 ± 0.277	0.775	0.101 ± 0.080	0.100 ± 0.089	0.807
8	0.347 ± 0.309	0.353 ± 0.354	0.755	0.118 ± 0.100	0.120 ± 0.117	0.712
9	0.332 ± 0.282	0.329 ± 0.283	0.795	0.116 ± 0.088	0.112 ± 0.088	0.429
10	0.311 ± 0.250	0.296 ± 0.230	0.190	0.104 ± 0.081	0.108 ± 0.078	0.464
11	0.284 ± 0.250	0.250 ± 0.241	0.003	0.098 ± 0.080	0.083 ± 0.074	0.006
12	0.247 ± 0.220	0.198 ± 0.200	< 0.001	0.085 ± 0.067	0.063 ± 0.066	< 0.001

*Bonferroni correction for multiple comparisons was applied. The *P* value considered to be significant was 0.0083 for SSD, and 0.0042 for AOD500/TISA500.

### Comparison of repeatability coefficients of anterior segment measurements between ANTERION and CASIAII

Both ANTERION and CASIAII showed small repeatability coefficients for measurements of ACA dimensions and anterior segment parameters. Whilst the repeatability coefficients of SSD and LV were similar between the instruments (p = 0.067 and P = 0.471, respectively), CASIAII showed slightly smaller but statistically significant different repeatability coefficients for ACD, PD, AOD500 and TISA500 than those measured by ANTERION (p ≤ 0.012) (Table 4).

**Table 4:** Comparison of repeatability coefficients of anterior segment parameters measured by ANTERION and CASIA II.

	ANTERION	CASIA II	P
	Repeatability coefficient (95% confidence interval)	Repeatability coefficient (95% confidence interval)	
Anterior chamber depth (mm)	0.017 (0.014 to 0.022)	0.016 (0.013 to 0.020)	< 0.001
Pupil diameter (mm)	0.398 (0.326 to 0.513)	0.371 (0.304 to 0.479)	0.012
Scleral spur-scleral spur distance (mm)	0.109 (0.089 to 0.140)	0.094 (0.077 to 0.121)	0.067
Lens vault (mm)	0.050 (0.041 to 0.065)	0.061 (0.050 to 0.079)	0.471
Angle opening distance 500 (mm)	0.042 (0.035 to 0.054)	0.035 (0.028 to 0.045)	< 0.001
Trabecular iris space area 500 (mm^2^)	0.016 (0.013 to 0.020)	0.014 (0.012 to 0.018)	0.001

## Discussion

The ANTERION and CASIAII represent the latest swept-source AS-OCT technology but they provide different ACA and anterior segment measurements. ANTERION measured smaller SSD, ACD and PD but greater AOD and TISA than CASIAII. The between-instrument agreement was poor; the spans of 95% limits of between-instrument agreement were ≥ 1.5-fold greater than the spans of within-instrument agreement (Fig. 1). Whereas both instruments showed relatively low test–retest variability, CASIAII had smaller repeatability coefficients for measurements of ACD, PD, AOD500 and TISA500 compared with ANTERION.

Although the ANTERION and CASIAII used similar light source (1300 nm and 1310 nm scan wavelength, respectively) and we applied similar scan protocols (6 B-scans for each eye, 768 A-scans/B-scan for ANTERION and 800 A-scans/B-san for CASIAII) to attain similar resolution (< 30 µm of transverse resolution) for anterior segment imaging, the two AS-OCT instruments show small but significant differences in the anterior segment parameters. The origin of the disparities is unclear but the fact that ANTERION measured smaller SSD (a horizontal dimension) as well as ACD (a vertical dimension) compared with CASIAII implies the disparities are likely to be systematic, which can be related to the differences in the calibration techniques and/or algorithms for segmentation of the anterior segment structures. The greater AOD/TISA obtained from ANTERION can be explained by its smaller PD measurement compared with CASIAII given the fact that AOD/TISA are negatively associated with the pupil size^12,13^. Although the anterior segment measurements obtained from ANTERION and CASIAII were highly correlated (R^2^ ranged between 0.866 to 0.998, Fig. 2), the finding that the spans of the 95% limits of agreement within the instrument were moderately to substantially smaller than those between the instruments suggests the two swept-source AS-OCT models cannot be used interchangeably for measurements of anterior segment parameters (Fig. 1).

We observed relatively small within-instrument test–retest variabilities for measurements of ACD, AOD500 and TISA500 for both swept-source AS-OCT instruments. It is interesting to note that although the differences in the within-instrument repeatability coefficients between ANTERION and CASIAII were small, significant differences in the repeatability coefficients of ACD, PD, AOD and TISA between the instruments were detected, which can be attributed to the very small test–retest variances for both AS-OCT devices. Whether the slightly smaller test–retest variabilities for CASIAII would translate to clinical benefits in the monitoring of ACA dimensions in patients with PACD and in the planning of anterior segment surgery remains to be determined.
